# Dual-Path Collaborative Transformer: Hybrid Attention and Stepwise Dilated Convolution for Remote Sensing Image Super-Resolution

**DOI:** 10.3390/s26175600

**Published:** 2026-09-03

**Authors:** Xiang Li, Min Zhang, Yuanhao Jin, Lixiang Xu, Bowen Wang, Jing Yang

**Affiliations:** 1 College of Artificial Intelligence and Big Data, Hefei University, Hefei 230601, China; 18021846026@163.com (X.L.); walikuatong@gmail.com (Y.J.); 15056379712@163.com (B.W.); yangjing@hfuu.edu.cn (J.Y.); 2 College of Artificial Intelligence Engineering, Hefei Institute of Technology, Hefei 238076, China; xulixianghf@163.com

**Keywords:** remote sensing, super-resolution, dual-path, deep learning, transformer

## Abstract

Remote Sensing Image Super-Resolution (RSISR) is a core task in geospatial image analysis. Convolutional neural networks (CNNs) have achieved significant breakthroughs in RSISR tasks by extracting local features. However, CNN-based methods struggle to capture long-range dependencies, thereby limiting SR performance. Recently, Transformer-based methods have demonstrated remarkable performance in capturing global information. Nevertheless, they remain inadequate for exploring high-frequency details and local features. To overcome these limitations, this work introduces a novel dual-path collaborative architecture, named DCTNet, which combines Transformer-based global modeling with convolution-driven local feature extraction. DCTNet is a hybrid network composed of a CNN-Transformer Residual Hybrid Group (CTHG). This group consists of two core components: the Dual-domain Fusion Window Attention Block (DFWAB) and the Stepwise Dilated Convolution (SDC). Specifically, the DFWAB incorporates channel and frequency attention mechanisms following the standard Transformer block to recover high-frequency details. Furthermore, by integrating stepwise dilated convolutions into the conventional Transformer architecture, the CTHG effectively captures both multi-scale local and global features. Additionally, we employ dense connections among the DFWAB modules to facilitate feature reuse across layers. Experimental results on the AID and UCMerced datasets demonstrate that DCTNet achieves competitive reconstruction performance across different scale factors, with statistically significant improvements observed in specific settings.

## 1. Introduction

Driven by continuous advances in remote sensing techniques, fine spatial resolution imagery has become indispensable in a wide range of practical applications, including object detection [1], scene recognition [2], environmental monitoring [3], and urban planning [4]. However, owing to hardware constraints and unfavorable imaging environments, obtaining remote sensing images with desirable spatial resolution remains a challenging task [5]. Therefore, RSISR aims to recover HR images from corresponding LR observations. It serves as an important technique for improving image resolution and promoting the practical application of remote sensing imagery.

SISR approaches are commonly divided into three categories: interpolation-based, reconstruction-based, and deep learning-based approaches [6,7,8,9,10]. In the early period, SR algorithms were mainly developed based on interpolation schemes, with bilinear and bicubic interpolation being two representative examples [11,12]. However, these methods are inherently limited in their ability to generate new high-frequency details, often resulting in over-smoothed edges and blurred regions. Reconstruction-based methods recover high-resolution images by solving an inverse problem, incorporating the effects of blur and noise from the image degradation process. These methods typically rely on image priors, such as smoothness, and leverage multiple low-resolution images for reconstruction. Recently, many studies have shifted their attention to deep learning frameworks, including CNNs and Transformers. CNN-based methods [8,13,14,15,16] perform well in local feature extraction. Despite their effectiveness, CNNs are constrained by the limited receptive fields of convolutional operators, making it challenging to establish long-distance feature interactions and thus reducing their ability to capture global context. Remote sensing images contain rich textures, complex structural information, and diverse object scales, such as vast farmlands and urban buildings. In particular, conventional CNN architectures may fail to fully exploit contextual information across remote sensing images, resulting in blurred boundaries in the reconstructed images.

Transformer-based methods [17,18,19,20,21,22] exhibit a strong ability to model global context through self-attention mechanisms. However, they often have difficulty reconstructing fine-grained local structures and high-frequency details, such as farmland textures, gully patterns, and building contours, because local pixel interactions are not sufficiently captured. Spatial correlations between neighboring pixels are crucial for restoring edges and fine textures in RSISR tasks. Furthermore, most existing approaches emphasize spatial-domain feature modeling, with frequency-domain cues often being overlooked. Although recent works have attempted to integrate frequency-domain features, such as the Fourier transform cross-attention layer in FocalSR [23], they often fail to effectively combine the spatial and frequency domains.

To mitigate the shortcomings of previous approaches, we present DCTNet, a novel dual-path collaborative Transformer network for RSISR that exploits the complementary strengths of CNNs and Transformers. DCTNet processes low-resolution inputs via two collaborative branches: a Transformer-based representation learning branch comprising multiple DFWABs and a CNN-based representation learning branch with SDC. DCTNet comprises two principal components: the Dual-domain Fusion Window Attention Block (DFWAB) and the Stepwise Dilated Convolution Block (SDC). Specifically, each DFWAB integrates channel and frequency-domain attention into the standard Transformer block to capture both global contextual dependencies and high-frequency structural details. Moreover, dense connections are introduced among successive DFWABs to promote hierarchical feature reuse, enhance information propagation and alleviate vanishing gradients. Complementarily, we employ SDC to capture local image features. By leveraging convolutional layers with varying dilation rates, this module effectively expands the receptive field without a substantial increase in parameters. Consequently, this design enables the effective extraction of textures and edges, which provide essential structural information in remote sensing images.

The effectiveness of DCTNet is verified through extensive experiments on standard remote sensing image benchmarks. Quantitative and qualitative comparisons indicate that DCTNet achieves competitive reconstruction performance across different datasets, scale factors, and evaluation metrics. The major contributions of this study are presented as follows:

(1) We propose DCTNet, a dual-path collaborative Transformer framework for RSISR. The proposed network combines the strengths of CNNs in local feature extraction with the capability of Transformers to capture global representations.
(2) We design a Dual-domain Fusion Window Attention Block (DFWAB), which integrates channel attention and frequency-domain attention into the standard Transformer block. This dual-domain mechanism enhances the interaction between spatial and spectral information, enabling the network to better recover high-frequency details and discriminative structural patterns. Additionally, dense connections are incorporated between successive DFWABs, promoting feature reuse and stabilizing feature propagation.
(3) We employ a Stepwise Dilated Convolution Block (SDC) to augment local feature extraction through an enlarged receptive field. By employing 3×3 convolutions with different dilation rates, SDC captures multi-scale textures and structural details without requiring explicit large-kernel convolutions.

Accordingly, the primary design objective of DCTNet is to strengthen reconstruction capability through complementary global, local, and frequency-domain feature modeling rather than to minimize model size or computational cost. The computational characteristics and practical limitations associated with this design choice are further discussed in Section 4.4.

## 2. Related Work

### 2.1. Image Super-Resolution

Early studies on SISR mainly adopted interpolation strategies, including nearest-neighbor, bilinear, and bicubic schemes [11,12]. However, such approaches are usually limited in recovering high-frequency components, which leads to overly smooth edges and blurred regions in the reconstructed images. Reconstruction-based methods typically rely on incorporating priors from HR images, such as gradient profiles [24] and edge smoothness priors [25]. Owing to the rapid evolution of deep CNNs, CNN-based approaches have shown strong reconstruction capability for SISR.

Dong et al. [8] made an early attempt to apply CNNs for image super-resolution by proposing SRCNN, which uses a three-layer CNN to establish an end-to-end transformation from LR images to their HR versions. Since then, a variety of SR models based on deep learning have emerged. Ledig et al. [26] introduced residual learning into SISR and proposed SRResNet, a deep residual architecture designed for image restoration. In the same study, they further developed SRGAN by incorporating a generative adversarial framework, where VGG-based perceptual features, content loss, and adversarial loss were jointly leveraged to enhance the perceptual fidelity of the reconstructed images. Lim et al. [27] proposed EDSR based on SRResNet by removing redundant components, including ReLU layers and BN layers, while introducing a residual scaling strategy to stabilize training. Subsequently, Wang et al. [28] further advanced SRGAN and introduced ESRGAN, which employs deeper residual-in-residual dense blocks instead of conventional residual blocks and incorporates a relativistic discriminator. Although CNN-based methods perform well in learning local image representations, their ability to capture long-range global context remains constrained.

In recent years, with the rapid progress of Transformer architectures in natural language processing [29,30,31], researchers have extended this architecture to computer vision tasks. Dosovitskiy et al. [17] first introduced Transformer architecture to computer vision tasks and proposed the Vision Transformer (ViT). In ViT, an image is partitioned into multiple two-dimensional patches, which are then flattened and transformed into embedding tokens. Liu et al. [32] presented the Swin Transformer, which limits self-attention computation to local windows and introduces shifted windows to enhance information exchange while maintaining lower computational complexity. Inspired by Swin Transformer, Liang et al. [19] proposed SwinIR for SISR, where residual Swin Transformer blocks are employed to model long-range contextual dependencies. To further address the high computational cost and limited non-local representation capability of window-based Transformer models, Zhang et al. [33] developed ELAN, which is designed to efficiently aggregate neighborhood structural information and non-local correlations through shifted convolution and multi-scale attention performed across feature groups. Following this line of research, Lu et al. [34] proposed ESRT, a compact and efficient super-resolution architecture that combines a lightweight CNN backbone with a Transformer backbone to obtain a favorable balance between reconstruction accuracy and computational efficiency. Overall, these Transformer-based SR methods exploit attention mechanisms to model long-range dependencies and pixel-wise similarities, thereby enhancing the representation capability of SR networks. Nevertheless, due to the limited explicit modeling of fine local textures, they may still face challenges in reconstructing high-frequency details and preserving delicate image structures.

### 2.2. Remote Sensing Image Super-Resolution

Remote sensing image super-resolution (RSISR) has attracted growing attention in recent years due to its importance in enhancing the visual quality and information content of satellite and aerial imagery. Early research mainly relied on sparse representation-based approaches. Pan et al. [35] developed an RSISR approach that combines compressive sensing, structural self-similarity, and dictionary learning. By exploiting recurrent structures within remote sensing images, this approach learns a dictionary for sparse representation of high-resolution patches and reconstructs high-resolution images within the compressive sensing framework. Nevertheless, such methods often have difficulty coping with the complexity and heterogeneity of remote sensing imagery, particularly in scenarios involving varying spatial resolutions and complex spatial patterns. With the advancement of deep learning techniques, a large number of SR networks have emerged. Lei et al. [36] introduced LGCNet, one of the pioneering deep learning frameworks for RSISR. By integrating local details with global contextual representations, LGCNet learns the residual relationship between LR and HR images, thereby improving the quality of reconstructed images. Subsequently, Meng et al. [37] proposed SRADSGAN, which integrates Generative Adversarial Networks (GANs) with stratified dense sampling and local-global attention mechanisms. In HSENet [38], self-similar patterns are captured through a single-scale similarity exploitation module, while cross-scale connections are employed to strengthen feature interaction among different image scales. Lei et al. [39] presented TransENet, an enhancement network based on the Transformer architecture, where Transformer representation learning is integrated with a staged enhancement strategy. Wang et al. [40] presented TSFNet, a spatial–frequency collaborative learning framework organized in two stages. The method mitigates geometric distortion and blurred fine details in large-scale RSISR by combining frequency-domain degradation representation with inter-stage feature integration. Chen et al. [41] proposed a recursive self-attention mechanism that aggregates features over multiple iterations, improving both the visual quality and texture preservation in SR tasks.

Although deep models have greatly advanced RSISR reconstruction, they still face numerous challenges related to scale diversity, complex background interference, and efficient network training. It is crucial to design a hybrid model to address the aforementioned issues.

## 3. Method

This section presents the proposed Dual-path Collaborative Transformer network (DCTNet). First, the overall architecture of DCTNet is introduced. Subsequently, two core components are described: the Dual-domain Fusion Window Attention Block (DFWAB) and the Stepwise Dilated Convolution (SDC) module.

### 3.1. Network Architecture

**Figure 1:**
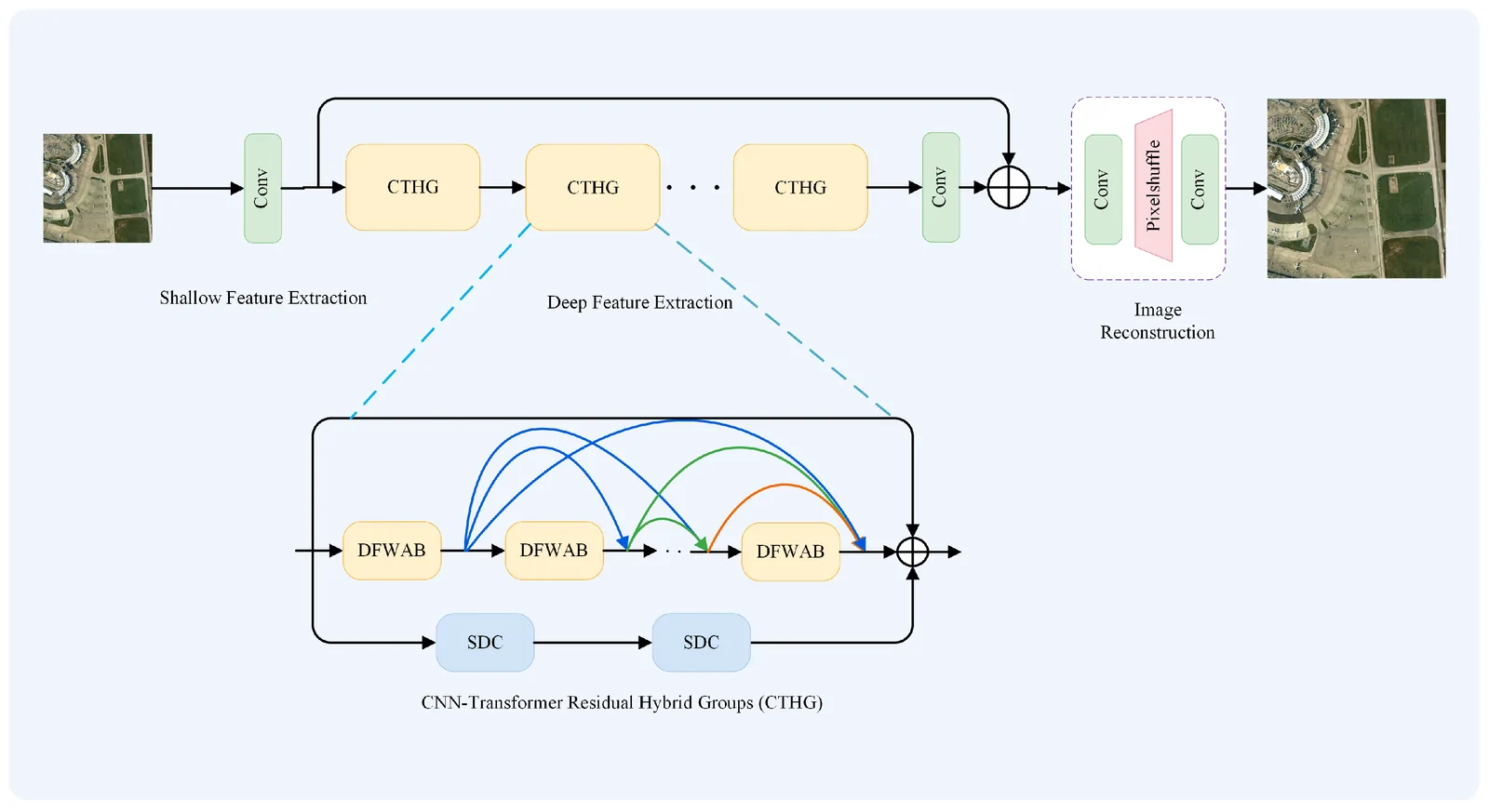
The overall architecture of the proposed network. The ellipses indicate repeated network blocks and do not represent omitted content.

The architecture of DCTNet is presented in Figure 1. It contains three major modules: a shallow feature extraction module, a deep feature extraction module, and an HR reconstruction module. Following the modular design commonly adopted in previous SR models [42,43,44], the proposed framework performs feature extraction and high-resolution image reconstruction in a structured manner. For an input LR remote sensing image $I_{\mathrm{LR}}\in R^{H\times W\times 3}$, a convolution operation is first applied to extract shallow features:

(1) $$F_{0}=\mathrm{Conv}\left(I_{\mathrm{LR}}\right),$$

where $F_{0}\in R^{H\times W\times C}$, *H* and *W* denote the height and width of the feature map, while *C* represents the channel dimension. Subsequently, the shallow features are fed into a stack of *N* CTHGs for deep feature extraction:

(2) $$F_{i}=H_{\mathrm{CTHG}}^{i}\left(F_{i-1}\right),\;i=1,2,\ldots ,N,$$

where $F_{N}$ represents the deep features extracted by the last CTHG block. Each CTHG is designed with residual learning and a collaborative CNN–Transformer dual-path structure, which facilitates the simultaneous representation of local structural patterns and long-range contextual dependencies in remote sensing imagery. Specifically, the CTHG block consists of Dual-domain Fusion Window Attention Blocks (DFWABs) and Stepwise Dilated Convolution Blocks (SDCs). Dense connections among the DFWABs facilitate continuous information propagation, promote feature reuse, and enhance the capability of the network to capture multi-scale local representations.

After deep feature extraction, a 3×3 convolutional layer is adopted to further refine and fuse the extracted features:

(3) $$F_{D}={\mathrm{Conv}}_{3\times 3}\left(F_{N}\right).$$

Then, a global residual connection is employed to merge the initial shallow representation with the enhanced deep features:

(4) $$F_{R}=F_{D}+F_{0}.$$

This residual learning strategy facilitates the preservation of low-frequency content while guiding the model to emphasize high-frequency detail recovery. Finally, the integrated features are passed through the reconstruction module, composed of convolutional layers and pixel-shuffle units, to generate the final super-resolved output:

(5) $$I_{\mathrm{SR}}=H_{\mathrm{Rec}}\left(F_{R}\right),$$

where $I_{\mathrm{SR}}\in R^{rH\times rW\times 3}$, and *r* denotes the upscaling factor.

### 3.2. Dual-Domain Fusion Window Attention Block (DFWAB)

**Figure 2:**
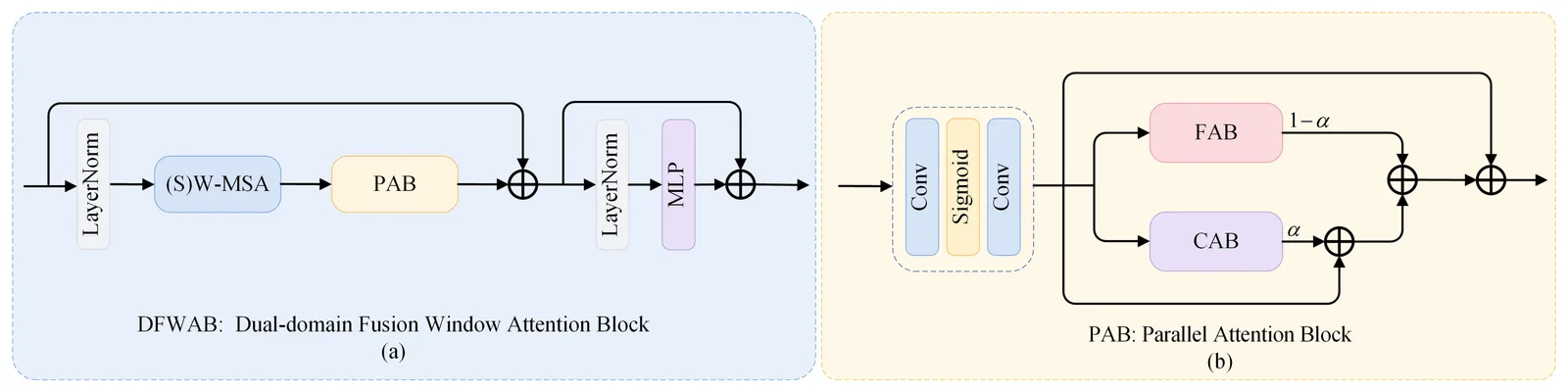
Structure and implementation details of the DFWAB in the CTHG. (**a**) Dual-domain Fusion Window Attention Block (DFWAB) and (**b**) Parallel Attention Block (PAB).

To overcome the limited receptive field of window-based attention and its limited ability to capture fine-grained details, we propose a Dual-domain Fusion Window Attention Block (DFWAB), as illustrated in Figure 2a. The DFWAB integrates a Parallel Attention Block (PAB) into the conventional Transformer block. As depicted in Figure 2b, PAB is constructed with two attention branches, namely the Channel Attention Block (CAB) and the Frequency Attention Block (FAB). Specifically, CAB is adopted to select important channels, while FAB extracts high-frequency features associated with edges, textures, and structural patterns, which are critical for high-quality image reconstruction. By jointly leveraging spatial, channel, and frequency information, DFWAB improves the network’s ability to model global dependencies while preserving fine local details.

To adaptively fuse the outputs of CAB and FAB, we introduce a learnable coefficient to balance their contributions. Furthermore, dense connections are incorporated among successive DFWABs to enable hierarchical feature reuse, facilitate information flow, and mitigate gradient degradation in deep networks. In this work, (S)W-MSA denotes the (Shifted) Window Multi-head Self-Attention mechanism, including W-MSA and its shifted-window variant (SW-MSA). The shifted window operation facilitates information exchange across neighboring windows while preserving the computational efficiency of local window-based self-attention. Therefore, given an input feature *X*, the operation of the DFWAB can be expressed as follows:

(6) $$\left\{\begin{array}{c} X_{I}=\mathrm{LN}\left(X\right)\\ X_{O}=\left(S\right)W-\mathrm{MSA}\left(X_{I}\right)\\ X_{P}=\alpha \mathrm{CAB}\left(X_{O}\right)+\left(1-\alpha \right)\mathrm{FAB}\left(X_{O}\right)\\ Y=\mathrm{MLP}\left(\mathrm{LN}(X_{P})\right)+X_{P} \end{array}\right.$$

where $X_{I}$ and $X_{O}$ denote the features processed by the LayerNorm and the (S)W-MSA module, respectively. $X_{P}$ represents the output feature maps of the PAB, and *Y* denotes the output of DFWAB. For an input feature map of resolution H×W with *C* channels, the feature map is first split into non-overlapping windows with a spatial size of N×N, after which self-attention is performed separately in each local window as follows:

(7) $$\mathrm{Attention}\left(Q,K,V\right)=\mathrm{SoftMax}\left(\frac{QK^{T}}{\sqrt{d}}+B\right)V$$

where *Q*, *K*, and *V* denote the query, key and value matrices, respectively, *d* refers to the feature dimension of the query and key, and *B* represents a bias term introduced to encode relative positional information.

**Figure 3:**
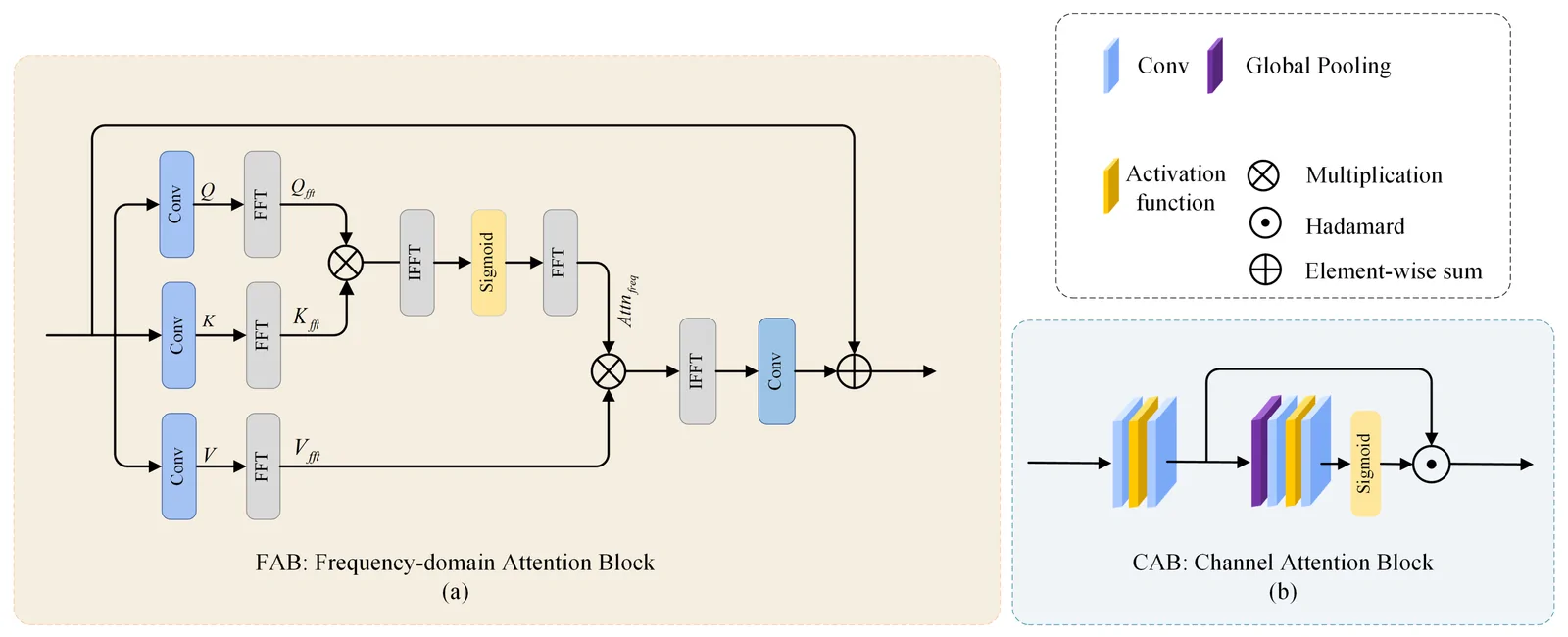
Structure of FAB and CAB. (**a**) Frequency-domain Attention Block (FAB) and (**b**) Channel Attention Block (CAB).

To address the insufficiency of spatial-domain attention in capturing high-frequency signals, we propose a Frequency-domain Attention Block (FAB). Specifically, the input features are initially mapped from the spatial domain to the frequency domain via the Fast Fourier Transform (FFT), where an attention mechanism is employed to enhance high-frequency detail reconstruction. As shown in Figure 3a, the FAB module consists of three fundamental components: the frequency-domain attention operation, FFT, and Inverse FFT (IFFT). Three independent 1×1 convolutional layers are employed to project the input feature map into three tensors: *Q*, *K*, and *V*.

Subsequently, these tensors are transformed into the frequency domain through the 2D FFT, resulting in $Q_{\mathrm{fft}}$, $K_{\mathrm{fft}}$, and $V_{\mathrm{fft}}$. The conjugate product of the query and key tensors is computed and normalized as follows:

(8) $${\mathrm{Attn}}_{freq}=\frac{Q_{\mathrm{fft}}\cdot \bar{K_{\mathrm{fft}}}}{\sqrt{H\times W}}$$

where ${\mathrm{Attn}}_{\mathrm{freq}}$ represents frequency-domain attention weights. These weights are mapped back to the spatial domain through the IFFT. A normalized attention map is then obtained by applying the Sigmoid activation. Subsequently, the normalized attention map is transformed back into the frequency domain. It is then multiplied by the value tensor $V_{fft}$. This operation is defined as follows:

(9) $${\mathrm{Output}}_{freq}={\mathrm{Attn}}_{freq}\cdot V_{fft}$$

where ${\mathrm{Output}}_{\mathrm{freq}}$ represents the frequency-domain features obtained after spectral reweighting. The IFFT is then applied to convert these features back into the spatial feature space. After passing through the output projection layer, the converted features are combined with the original input through a residual connection to produce the module output. This design allows the model to capture high-frequency spectral information while maintaining the integrity of the original feature representation.

The Channel Attention Block, illustrated in Figure 3b, is designed to refine channel-level feature representations by learning dependencies among different channels. To address the computational complexity introduced by high-dimensional channel features, a reduction ratio *r* is adopted in the two convolutional layers. Given an input feature with *C* channels, the first convolutional layer compresses the channel dimension to $\frac{C}{r}$, and the second layer projects the features back to *C* channels, yielding the transformed feature *F*. Subsequently, a channel attention unit is applied to *F*. First, spatial features are summarized by global average pooling to form a compact channel descriptor. This descriptor is refined by two sequential 1×1 convolutions with ReLU between them, after which a sigmoid function produces the channel-wise attention coefficients. These weights are multiplied with *F* via a Hadamard product to adaptively recalibrate the channel responses. The channel attention mechanism in CAB is computed according to the following formulation:

(10) $$\left\{\begin{array}{c} F={\mathrm{Conv}}_{3\times 3}\left(\phi \left({\mathrm{Conv}}_{3\times 3}\left(x\right)\right)\right),\\ \mathrm{Attn}\left(F\right)=\sigma \left({\mathrm{Conv}}_{1\times 1}\left(\delta \left({\mathrm{Conv}}_{1\times 1}\left(\mathrm{GAP}(F)\right)\right)\right)\right),\\ \mathrm{CAB}(x)=F⊙\mathrm{Attn}(F). \end{array}\right.$$

where ϕ(·) denotes the GELU activation function, δ(·) denotes the ReLU activation function, σ(·) denotes the Sigmoid function, GAP(·) denotes global average pooling, and ⊙ represents the Hadamard product. During the fusion process of the FAB and CAB branches, a learnable coefficient α is introduced to flexibly modulate the contribution ratio between the CAB and the FAB. The coefficient is optimized using gradient descent. This design allows the network to learn an appropriate balance between the two attention branches during optimization, leading to more effective integration of channel- and frequency-domain information.

### 3.3. Stepwise Dilated Convolution (SDC)

**Figure 4:**
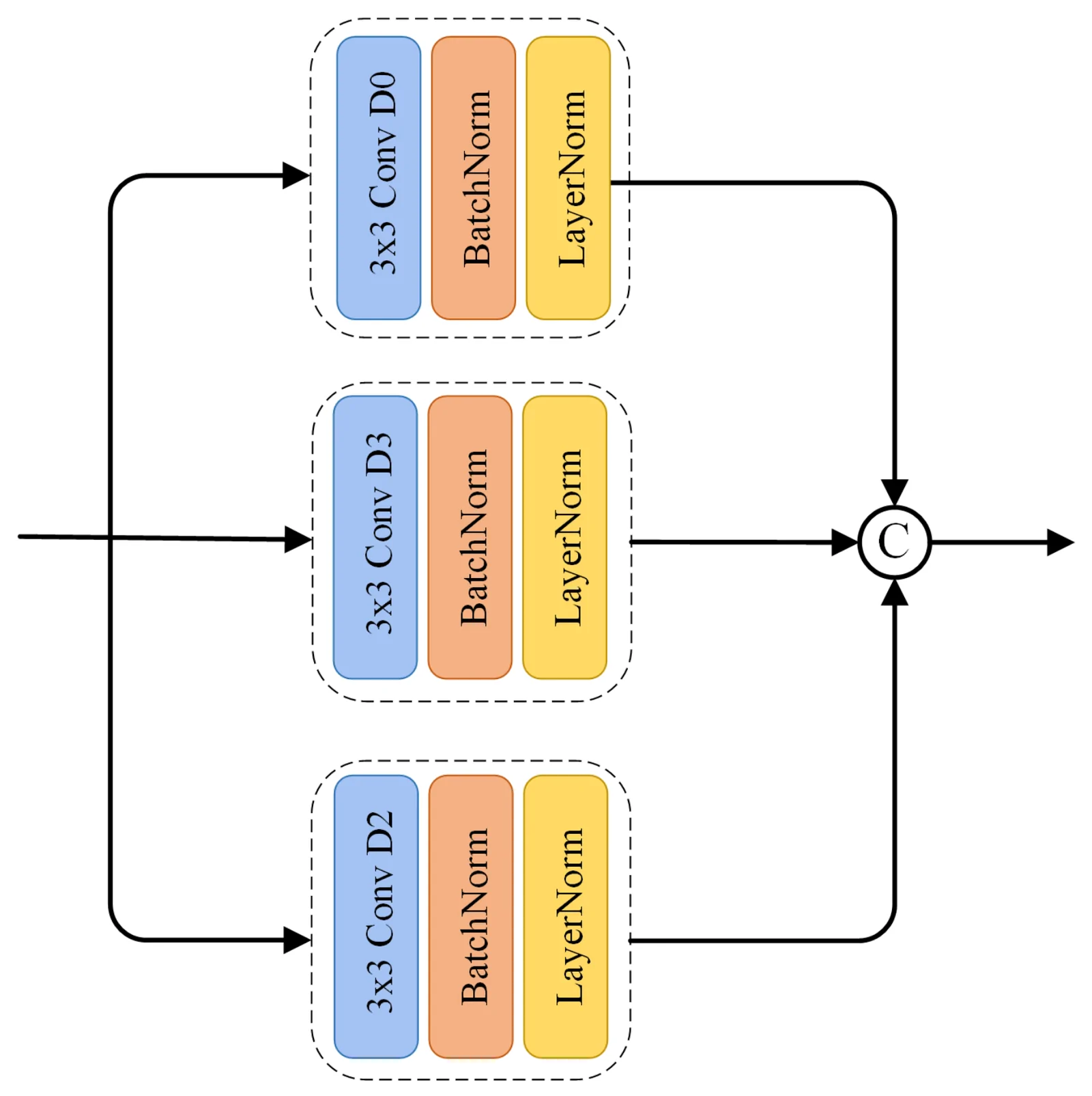
Structure and implementation principle of the SDC module. C denotes feature concatenation.

To more effectively exploit image features at multiple scales, we design the Stepwise Dilated Convolution Block, as shown in Figure 4. For multi-level contextual modeling, three parallel 3×3 convolutional paths configured with distinct dilation rates are adopted. Specifically, the path with a dilation rate of 1 is responsible for extracting fine local details, whereas the paths with dilation rates of 2 and 3 expand the effective receptive field to encode medium-range texture cues and broader contextual dependencies, respectively. The outputs of different paths are concatenated along the channel dimension and subsequently compressed by a 1×1 convolution to produce a compact multi-scale representation. This design establishes a pyramid-like receptive field corresponding to 3×3, 5×5 and 7×7 kernels, thereby expanding the receptive range for feature extraction without incurring the additional parameter size and computational cost associated with large convolution kernels. By integrating features from different receptive fields, the proposed SDC module enhances the representation of complex textures and structural details in super-resolved images. The standard convolutional branch preserves fine local responses, while the dilated convolutional branches introduce wider contextual cues for modeling texture patterns and structural dependencies. Through the subsequent channel-wise concatenation and 1×1 convolutional fusion, these complementary features are effectively aggregated, thereby improving the DCTNet’s capability to reconstruct complex textures, edges, and image structures.

## 4. Experiments

### 4.1. Experimental Datasets and Evaluation Settings

To verify the effectiveness of the proposed model, we conducted experiments on two widely used public remote sensing benchmarks, namely UCMerced [45] and AID [46]. The characteristics of these two datasets are described in detail below:

(1) The UCMerced dataset contains 21 categories, such as agricultural, buildings, chaparral, and river. Each category contains 100 images. In our experiments, 80% of the images were used for model training, while the remaining 20% were adopted for testing. Additionally, 30 images were randomly chosen from the training split to construct the validation set.
(2) The AID dataset consists of 10,000 images covering 30 categories, including airport, bridge, commercial, desert, pond, and railway station. For a fair comparison, the dataset was split following the same setting, where 80% of the samples were assigned to the training set and the rest were used for testing. Meanwhile, 150 samples were randomly drawn from the training data as the validation set. The quantitative results were reported in terms of PSNR and SSIM, both evaluated on the Y-channel of the YCbCr color space.

The same split indices were used for seeds 0–4 to ensure that the observed variability was attributable to model training rather than data resampling.

### 4.2. Implementation Details

#### 4.2.1. Experimental Setup

The proposed method was evaluated with upscaling factors of ×2, ×3, and ×4. During the training stage, an exponential moving average (EMA) strategy was employed to enhance the stability of optimization. In the DCTNet, the numbers of CTHG and DFWAB units were both set to 6, while the number of SDC units in each CTHG was set to 2. The channel dimension was fixed at 180. For the (S)W-MSA component, the attention head number was configured as 6, and the window size was fixed at 16. Regarding the hyperparameters of the proposed modules, the learnable coefficient in PAB was initialized as 0.5, and the channel-reduction ratio *r* in CAB was set to 3. The network was trained using the $L_{1}$ reconstruction loss, which minimizes the absolute difference between the reconstructed SR image and the corresponding HR ground-truth image. For optimization, the network was trained with the Adam optimizer [47], where $\beta_{1}=0.9,\;\beta_{2}=0.99$ and $\epsilon =10^{-8}$. The learning rate was initially set to 2 × 10^−4^, and the batch size was set to 4. The training budget varied with the dataset and upscaling factor: AID ×2 and ×3 were trained for 500,000 iterations each, AID ×4 for 250,000 iterations, and all UCMerced settings for 120,000 iterations. For all settings, the learning rate was halved at 50%, 80%, 90%, and 95% of the total training iterations. The proposed approach was implemented using PyTorch 2.5.1 [48] and all experiments were conducted on an NVIDIA GeForce RTX 3090 GPU.

#### 4.2.2. Statistical Analysis

To evaluate training variability, we trained DCTNet, HAT, and MFG-HMoE five times each with random seeds 0–4 for ×3 and ×4 upscaling. Weight initializations were randomized across runs. All other settings—including dataset splits, preprocessing, hyperparameters, checkpoint selection, and evaluation—were fixed. Results are reported as the mean and sample standard deviation over five runs.

Two-sided Welch’s t-tests were conducted on the PSNR and SSIM values obtained from each independent run. For each dataset and scale factor, DCTNet was evaluated against the most competitive baseline selected from the comparison set: HAT for AID ×3 and UCMerced ×3, and MFG-HMoE for AID ×4 and UCMerced ×4. The mean difference (DCTNet—baseline) was reported with two-sided 95% confidence intervals. To control the family-wise error rate, *p*-values from the eight dataset–scale–metric comparisons were adjusted using the Holm procedure.

### 4.3. Ablation Study

To investigate the effects of CAB, FAB, and SDC on model performance, ablation studies were conducted on the UCMerced and AID datasets at an upscaling factor of ×4. The obtained results are summarized in Table 1 and Table 2.

With SDC enabled, the configuration using CAB and SDC without FAB achieved 28.92 dB/0.7874 on UCMerced and 30.89 dB/0.8154 on AID, whereas the configuration with FAB and SDC but without CAB obtained 28.95 dB/0.7888 and 30.93 dB/0.8168, respectively. These results indicate that FAB provides a larger numerical contribution than CAB under otherwise identical configurations on both datasets. The complete DCTNet configuration achieves the best performance, reaching 28.97 dB/0.7892 on UCMerced and 30.99 dB/0.8177 on AID. Compared with the FAB–only setup, adding CAB further improves PSNR by 0.02 dB and 0.06 dB on UCMerced and AID, respectively. This consistent trend suggests that channel-domain information provides complementary cues to the frequency-domain representation.

A learnable fusion coefficient α is introduced in PAB to adaptively balance the two attention branches. To assess sensitivity to initialization, we tested four initial values of α, as shown in Table 3. Among these, initialization at α=0.5 achieves the best reconstruction performance, with 28.97 dB in PSNR and 0.7892 in SSIM. This result indicates that a symmetric initialization provides a favorable starting point for optimization. Note that α remains learnable throughout training. Thus, 0.5 represents the initialization rather than a fixed or final fusion ratio. To further examine whether the fusion coefficient is effectively optimized, we tracked its evolution during training under different datasets and scaling-factor settings.

**Figure 5:**
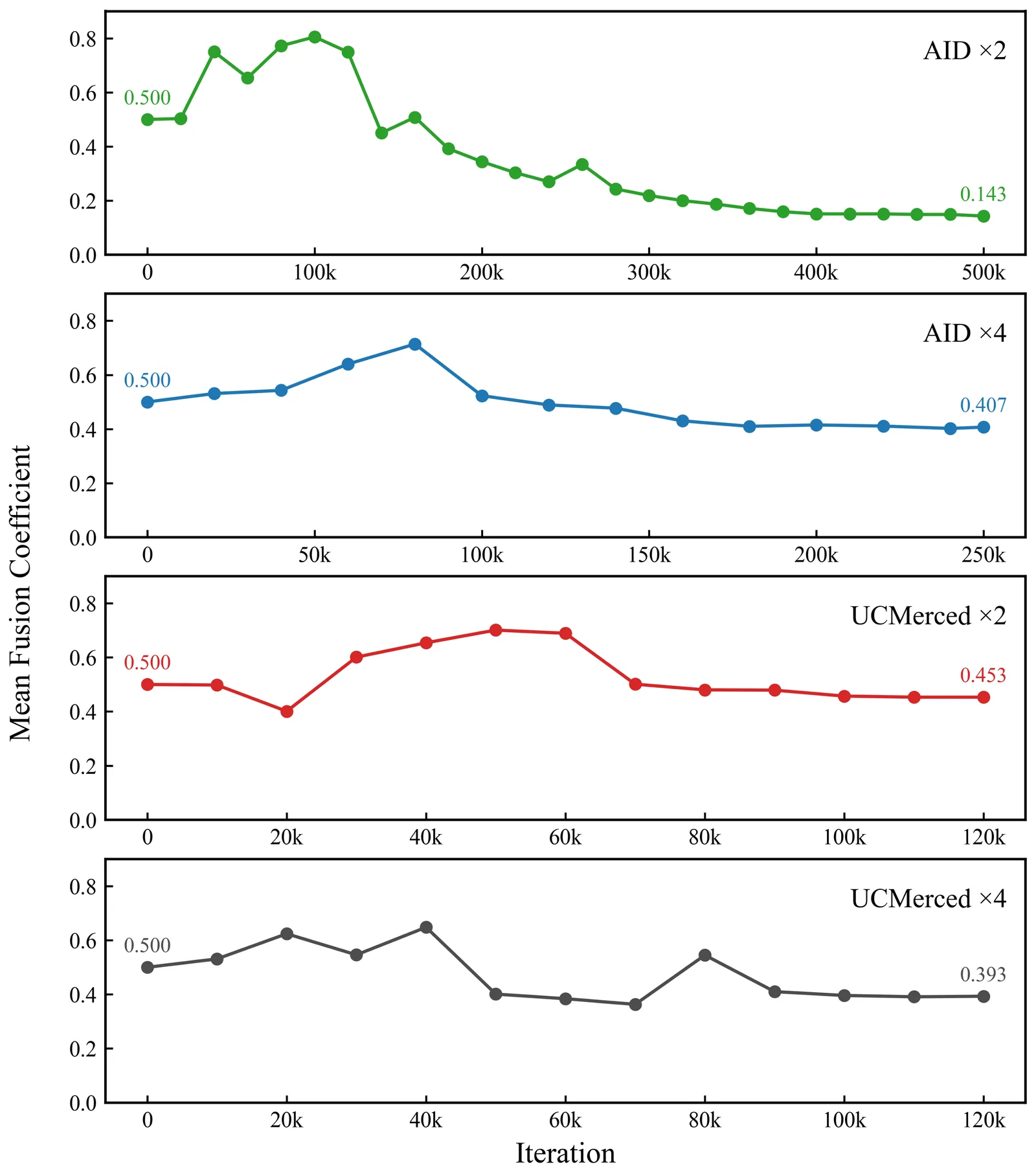
Evolution of the mean learnable fusion coefficient during training under different datasets and scaling-factor settings.

As illustrated in Figure 5, we calculated the mean of the 36 learnable fusion coefficients at selected training checkpoints. Although all coefficients were initialized to 0.5, their mean values deviate substantially from this initial state during training across all four settings. For AID ×2, the mean coefficient first increases to approximately 0.805 in the early training stage and then decreases progressively to 0.143 at the final checkpoint. A similar adaptive adjustment is observed for AID ×4 and UCMerced ×2, where the coefficients initially increase and subsequently stabilize at approximately 0.407 and 0.453, respectively. For UCMerced ×4, although a relatively pronounced fluctuation occurs during the intermediate stage, the mean coefficient becomes stable near 0.39 toward the end of training. These non-monotonic trajectories indicate that the fusion coefficients are actively optimized rather than remaining close to their initial value. The final mean coefficients also vary across the four experimental settings, suggesting that the relative contributions of the two attention branches are adaptively adjusted under different dataset–scale configurations. Notably, all final mean coefficients are below 0.5, indicating a relatively greater contribution from the frequency-attention branch under the adopted fusion mechanism. This tendency is consistent with the ablation results, in which FAB provides larger numerical improvements than CAB under otherwise identical configurations. The differences among the final coefficient values further suggest that the learned fusion is sensitive to the characteristics of the specific reconstruction setting rather than converging to a universal weighting pattern. Although the current observations do not support attributing these variations to a single factor such as texture density or structural regularity, they demonstrate that the learnable coefficient provides a flexible mechanism for adapting the balance between channel- and frequency-domain information across different reconstruction conditions.

### 4.4. Comparison with Other Methods

We further compared DCTNet with several SR approaches, namely, SRCNN [8], SwinIR [19], ELAN [33], HSENet [38], TransENet [39], HAT [49], DRCT [50], MFG-HMoE [51], and MambaIRv2-S [52]. For consistency and fairness, each comparison model was retrained based on its publicly available code and tested under the same evaluation protocol as the proposed method for scale factors of ×2, ×3 and ×4.

Table 4 shows that DCTNet remains within the top-performing group on both datasets, although its relative ranking varies with scale factors and metrics. To evaluate the stability and effectiveness of the method, we conducted five independent runs for DCTNet, HAT, and MFG-HMoE, as reported in Table 5, Table 6 and Table 7.

On AID ×3, DCTNet shows small positive mean differences of 0.0102 dB in PSNR and 0.000202 in SSIM relative to HAT, but neither difference is statistically significant after Holm correction. On AID ×4, DCTNet shows a statistically significant PSNR improvement of 0.0218 dB over MFG-HMoE (Holm-adjusted *p* = 0.0089), whereas its SSIM is lower by 0.000616 and the difference is not statistically significant. On UCMerced ×3, the PSNR difference of +0.0312 dB is not statistically significant, while DCTNet has a statistically significant SSIM decrease of 0.002022 relative to HAT (Holm-adjusted *p* = 0.0046). On UCMerced ×4, DCTNet significantly improves PSNR by 0.0500 dB and SSIM by 0.003094 over MFG-HMoE (both Holm-adjusted *p*-values < 0.001). These results demonstrate that the model achieves competitive performance, with statistically significant gains observed under specific experimental settings.

Table 8 summarizes the class-wise PSNR results on the AID test set for ×4 super-resolution. The results show that DCTNet maintains numerically competitive reconstruction performance across a wide range of scene categories, with only modest differences observed among several of the top-performing methods. These class-wise results are presented to illustrate how the reconstruction performance varies across different scene types rather than to establish category-specific superiority. Since no repeated-run statistical tests or multiple-comparison corrections were conducted at the individual class level, the numerical differences in Table 8 should be interpreted descriptively. Overall, the results indicate that the performance of DCTNet is relatively consistent across diverse remote-sensing scenes, while no statistical inference is made regarding its superiority within any particular category.

Table 9 compares the model complexity and inference efficiency of the competing SR methods. All efficiency measurements were performed on 128×128 LR input images with a batch size of 1. The results show that DCTNet incurs a relatively high computational cost compared with most of the competing approaches, which represents a limitation for resource-constrained deployment. This overhead mainly arises from the joint modeling of spatial-domain, frequency-domain, and multi-scale representations. Accordingly, DCTNet is primarily designed to enhance reconstruction capability rather than to minimize computational complexity, and it should not be regarded as a lightweight or real-time SR architecture. The current model is therefore more suitable for offline or server-side remote-sensing image enhancement scenarios in which reconstruction quality is prioritized over inference efficiency.

**Figure 6:**
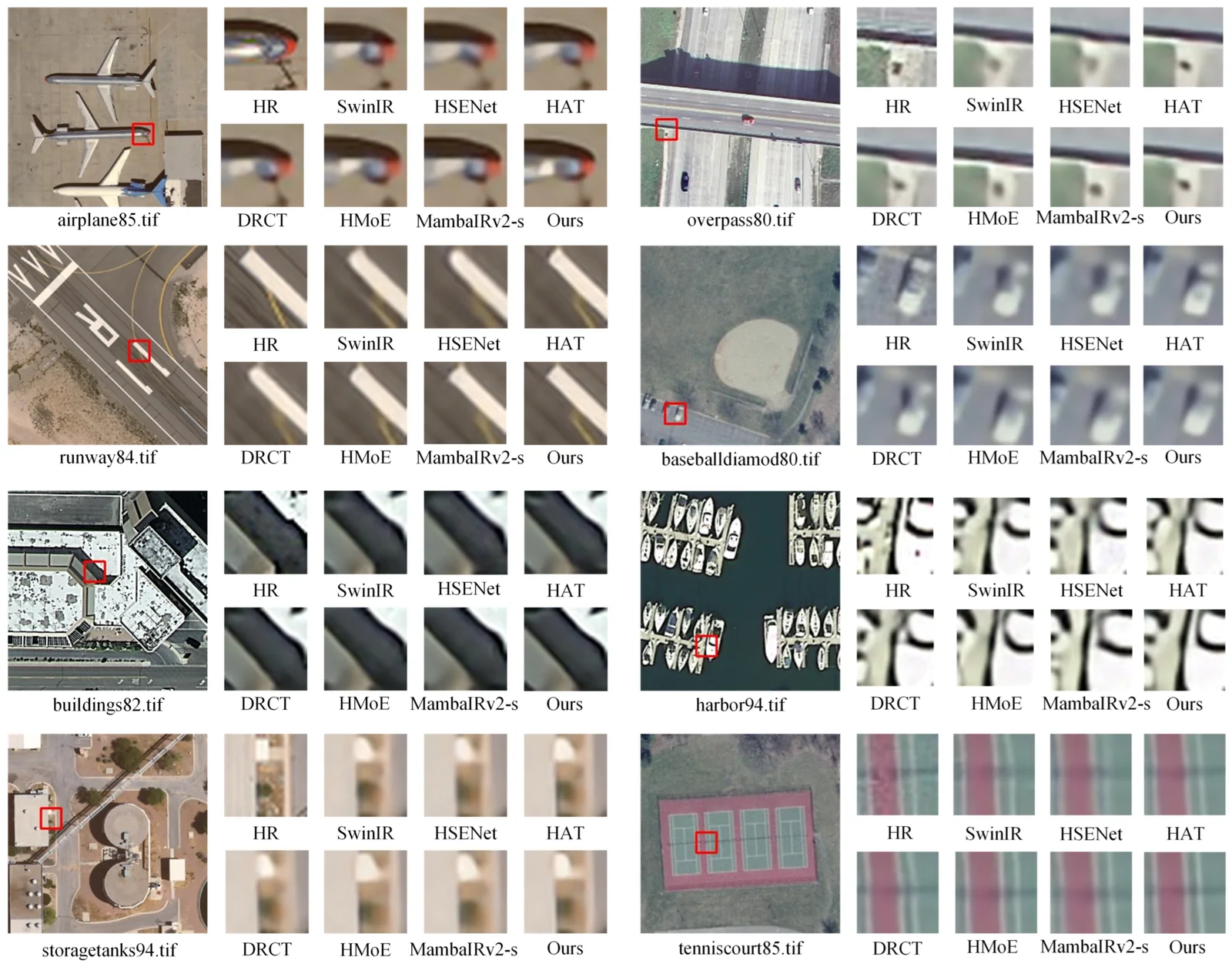
Visual comparisons for ×4 super-resolution on the UCMerced dataset. The parts used for comparison are marked with red boxes in the original images.

To qualitatively evaluate the reconstruction quality, Figure 6 compares DCTNet with six competing methods using representative UCMerced samples, such as “overpass80”, “airplane85”, and “runway84”. The enlarged regions marked by red boxes are provided to facilitate a detailed comparison of local reconstruction quality among different methods. It can be observed that most existing methods can recover the overall image structure to some extent. However, they still suffer from blurred edges, over-smoothed textures, and insufficient restoration of fine-grained details. For example, in the airplane and overpass scenes, the results produced by competing methods exhibit noticeable boundary ambiguity around aircraft components and road markings, whereas the proposed DCTNet reconstructs sharper contours and more distinguishable structural details. In the runway and building scenes, DCTNet better preserves linear structures and edge continuity, thereby reducing the distortion and smoothing artifacts observed in other methods. For texture-rich scenes, such as harbor and tennis court, the proposed method generates clearer local patterns and more faithful high-frequency details, indicating its stronger capability to recover complex spatial information in remote sensing images.

**Figure 7:**
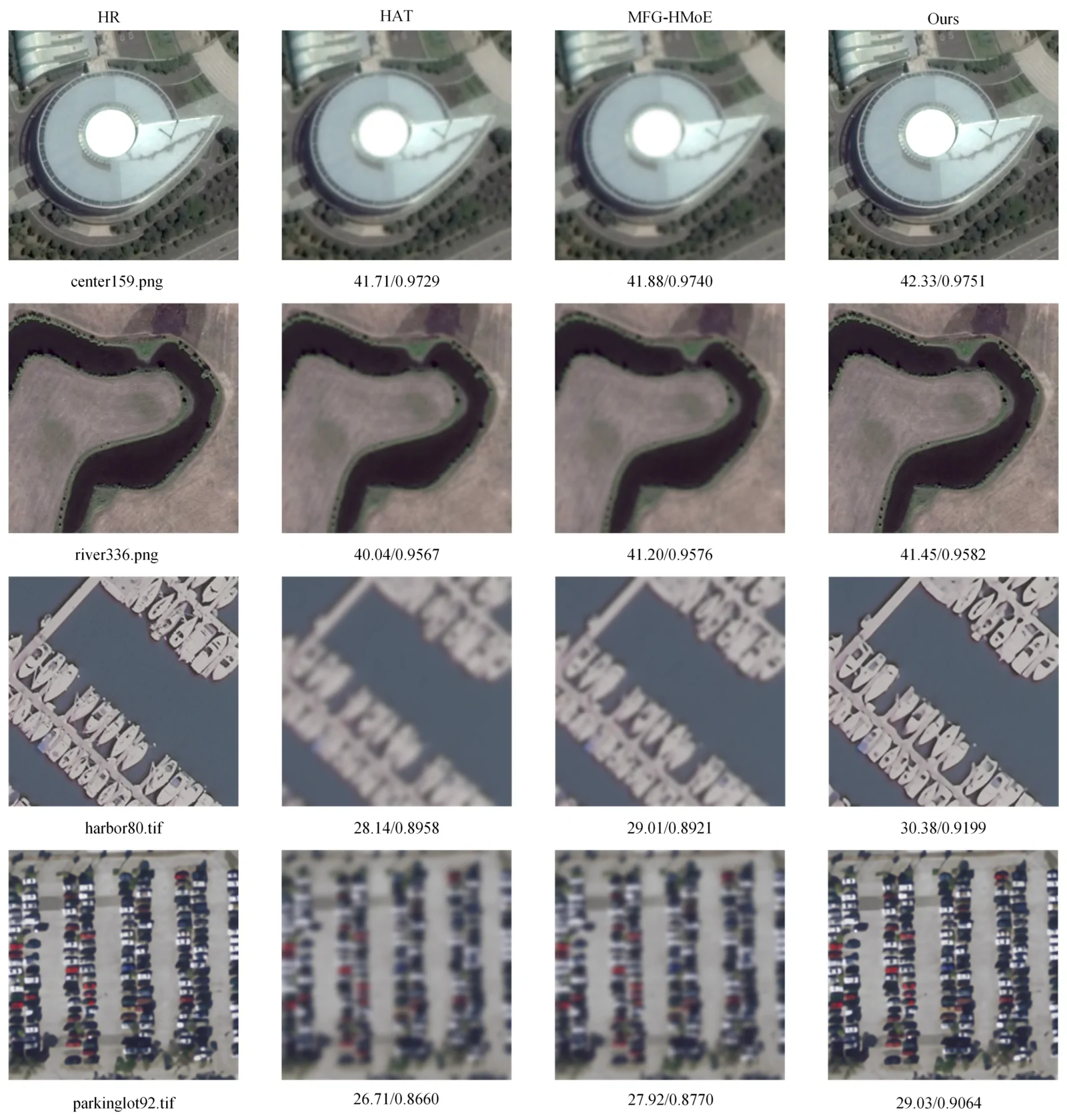
Quantitatively guided visual comparisons for ×4 super-resolution on the AID and UCMerced datasets.

Using fixed models and the same settings, we computed image-level PSNR/SSIM for each test image in the AID and UCMerced datasets under ×4 SR, following the Y-channel protocol from our quantitative experiments. With MFG-HMoE as the baseline, we calculated ΔPSNR and ΔSSIM per image. Images with gains in both metrics were ranked by ΔPSNR, and the top two from each dataset were selected while ensuring scene diversity. Figure 7 shows center/river scenes from AID and harbor/parking-lot scenes from UCMerced, with image-level PSNR/SSIM values below each image. For the four examples, DCTNet yields higher image-level PSNR/SSIM values than HAT and MFG-HMoE and reconstructs clearer contours and more distinguishable repetitive structures, particularly in the harbor and parking-lot scenes.

**Figure 8:**
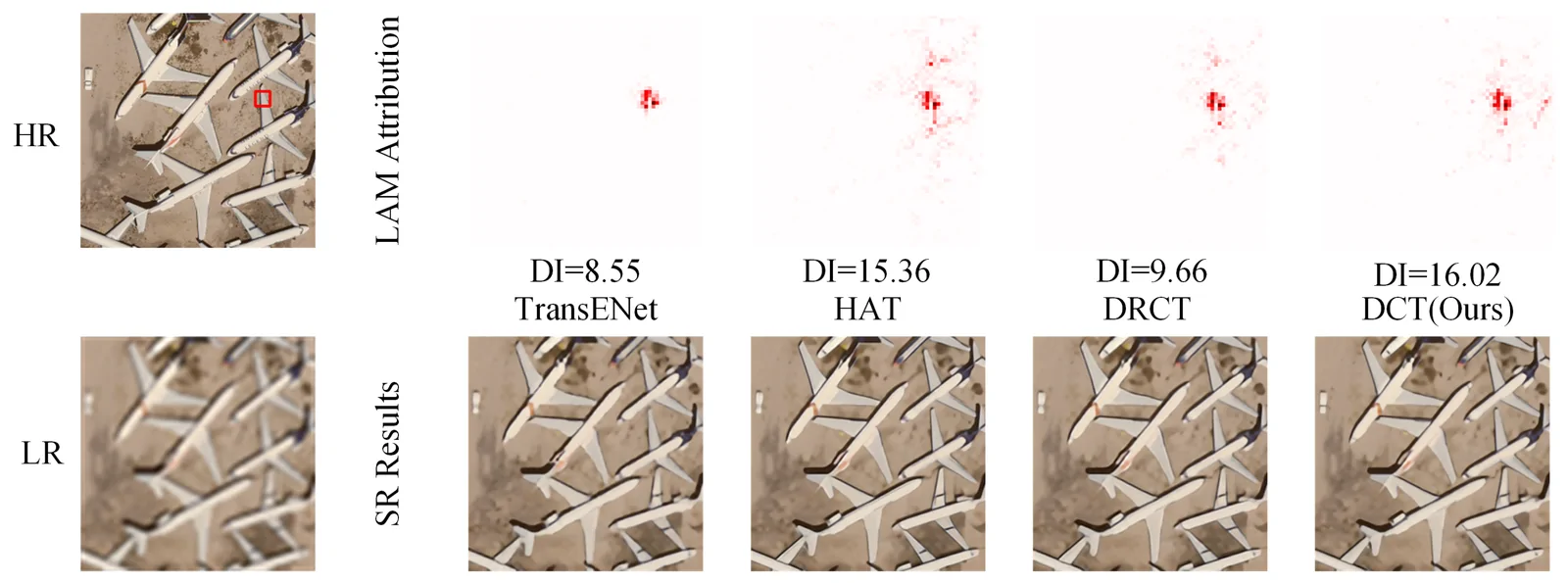
LAM results for different models. The red box indicates the target region used for the LAM analysis.

In addition to the qualitative and quantitative comparisons presented in Figure 6 and Figure 7, we further conducted an interpretability analysis using the Local Attribution Map (LAM) [53] to investigate how different methods utilized information from the low-resolution input during reconstruction. As shown in Figure 8, the LAM results and the corresponding Diffusion Index (DI) values are presented for several representative SR methods. The DI characterizes the spatial extent of the input pixels contributing to the reconstructed region, with a larger value indicating a broader effective receptive field. DCTNet exhibits the highest DI value among the compared methods. Specifically, its DI reaches 16.02, indicating that a broader spatial region of the LR input contributes to the reconstruction of the target area in the analyzed example. Compared with the more localized attribution patterns of the other methods, DCTNet shows a wider and more coherent distribution of contributing pixels. It should be noted, however, that DI characterizes the spatial extent of information utilization rather than the structural fidelity of the reconstructed image. A broader attribution range therefore does not necessarily lead to a higher SSIM, because the effectiveness of contextual information also depends on how well long-range cues are coordinated with local structural details. This behavior is reflected in the UCMerced ×3 results, where the broader spatial attribution observed for DCTNet does not translate into a higher SSIM than HAT. One possible explanation is that incorporating information from a wider spatial region may benefit contextual modeling while providing limited or even less favorable contributions to local structural preservation under certain scale settings. Therefore, the LAM results should be interpreted as evidence that DCTNet is capable of exploiting a broader spatial context during reconstruction, rather than as evidence of consistently improved structural similarity across all experimental conditions.

**Figure 9:**
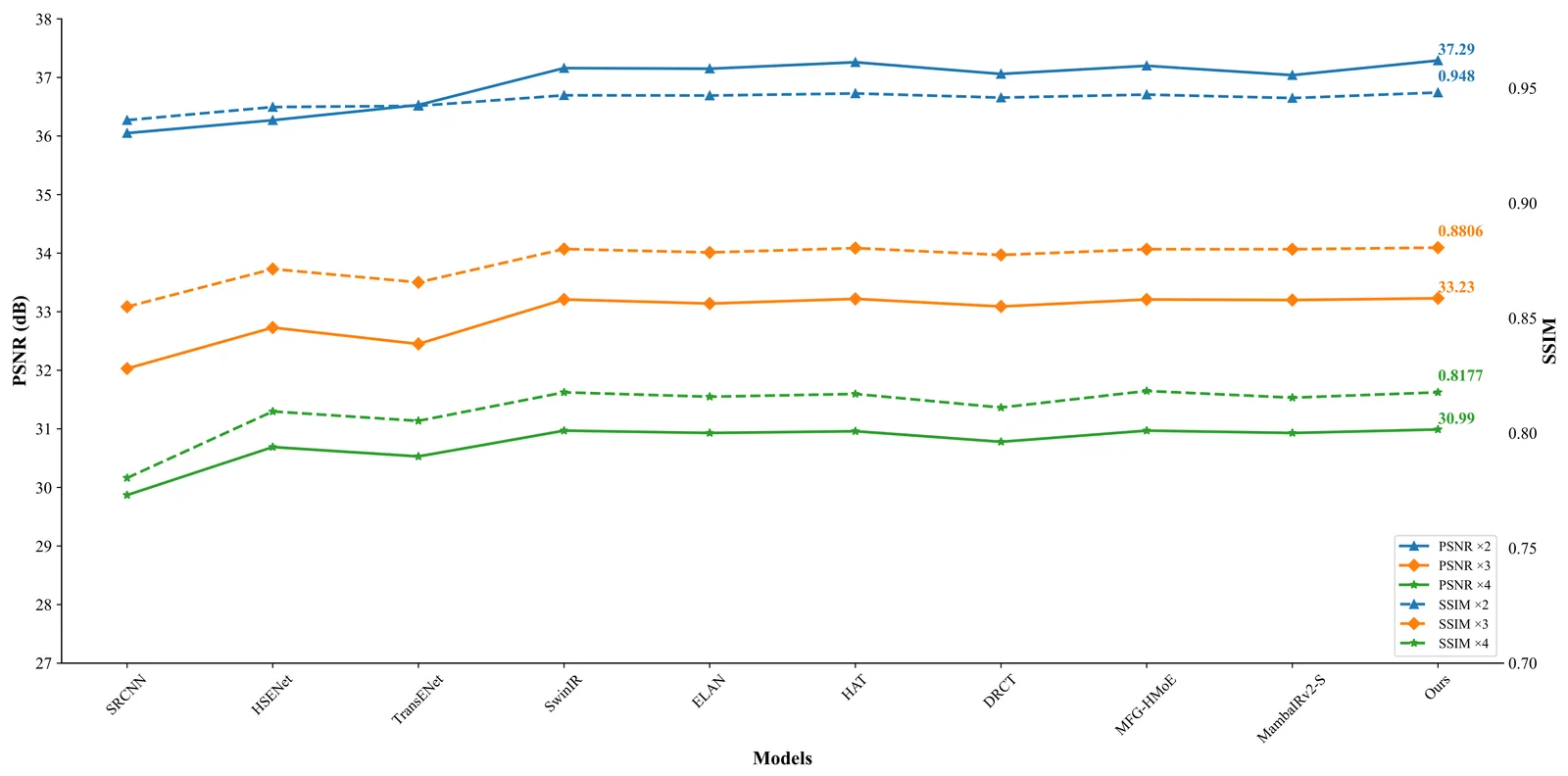
PSNR and SSIM comparison on the AID dataset for scale factors ×2, ×3, and ×4.

**Figure 10:**
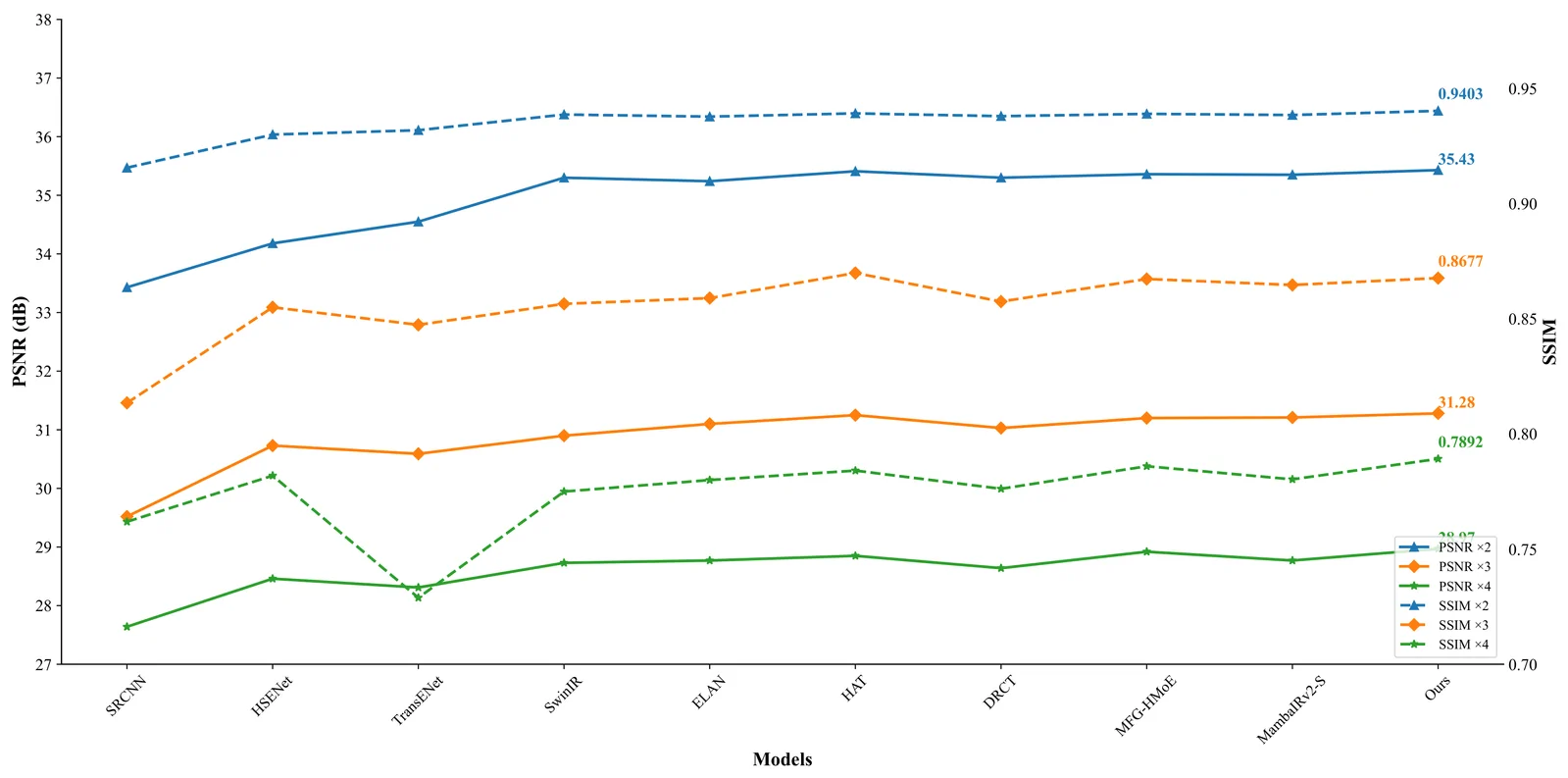
PSNR and SSIM comparison on the UCMerced dataset for scale factors ×2, ×3, and ×4.

The LAM analysis provides complementary evidence regarding the spatial extent of information utilization during reconstruction. Figure 9 and Figure 10 further provide a descriptive visualization of the quantitative results reported in Table 4. Overall, DCTNet remains among the top-performing methods across the two datasets and scale factors, although its relative ranking varies with the evaluation metric and experimental setting.

## 5. Conclusions

This work introduces DCTNet, a dual-path framework for RSISR. The proposed DFWAB adaptively integrates channel and frequency-domain information, while the SDC module enlarges the receptive field for local structural modeling. Dense connections further facilitate feature reuse and information propagation. Experiments on the AID and UCMerced datasets show that DCTNet remains among the top-performing RSISR methods overall. However, its relative performance varies across datasets, upscaling factors, and evaluation metrics. Five-run analyses at ×3 and ×4 further confirm this setting-dependent behavior. DCTNet achieves a statistically significant PSNR improvement on AID ×4 and significant improvements in both PSNR and SSIM on UCMerced ×4. In contrast, neither PSNR nor SSIM shows a significant difference on AID ×3, and the SSIM difference on AID ×4 is also not statistically significant. On UCMerced ×3, DCTNet shows no significant PSNR improvement and has a significantly lower SSIM than HAT. Overall, these results indicate that DCTNet provides competitive reconstruction performance, although its advantages are not consistent across all experimental settings. In addition, the relatively high computational cost limits its applicability in resource-constrained or real-time deployment scenarios. Therefore, the current architecture is better suited to offline or server-side remote-sensing image enhancement tasks, where reconstruction quality is prioritized over inference efficiency.

## Figures and Tables

**Table 1:** Fine-grained ablation study of CAB, FAB, and SDC on the UCMerced dataset for ×4 super-resolution.

CAB	FAB	SDC	PSNR	SSIM
×	×	×	28.89	0.7867
×	×	✓	28.91	0.7870
✓	✓	×	28.94	0.7874
✓	×	✓	28.92	0.7874
×	✓	✓	28.95	0.7888
✓	✓	✓	**28.97**	**0.7892**

Note: ✓ indicates that the corresponding module is included, whereas × indicates that it is excluded. Bold values indicate the best performance.

**Table 2:** Fine-grained ablation study of CAB, FAB, and SDC on the AID dataset for ×4 super-resolution.

CAB	FAB	SDC	PSNR	SSIM
×	×	×	30.84	0.8092
×	×	✓	30.87	0.8131
✓	✓	×	30.91	0.8146
✓	×	✓	30.89	0.8154
×	✓	✓	30.93	0.8168
✓	✓	✓	**30.99**	**0.8177**

Note: ✓ indicates that the corresponding module is included, whereas × indicates that it is excluded. Bold values indicate the best performance.

**Table 3:** Influence of different initialization values of the learnable fusion coefficient α in PAB.

α	0.1	0.3	0.5	0.7
**PSNR**	28.94	28.92	**28.97**	28.94
**SSIM**	0.7881	0.7874	**0.7892**	0.7873

Note: Bold values indicate the best performance.

**Table 4:** Quantitative results on the AID and UCMerced test sets. The best and second-best performance values are marked in red and blue, respectively.

Dataset	Metric	SRCNN	HSENet	TransE Net	SwinIR	ELAN	HAT	DRCT	MFG-HMoE	MambaIR v2-S	Ours
AID	PSNR	36.05	36.27	36.53	37.16	37.15	37.26	37.06	37.20	37.04	37.29
×2	SSIM	0.9360	0.9417	0.9422	0.9468	0.9467	0.9476	0.9458	0.9471	0.9456	0.9480
AID	PSNR	32.03	32.73	32.45	33.21	33.14	33.22	33.09	33.21	33.20	33.23
×3	SSIM	0.8549	0.8713	0.8655	0.8800	0.8785	0.8804	0.8774	0.8799	0.8799	0.8806
AID	PSNR	29.87	30.69	30.53	30.97	30.93	30.96	30.78	30.97	30.93	30.99
×4	SSIM	0.7805	0.8094	0.8053	0.8177	0.8158	0.8170	0.8111	0.8183	0.8154	0.8177
UCM	PSNR	33.43	34.18	34.55	35.30	35.24	35.41	35.30	35.36	35.35	35.43
×2	SSIM	0.9156	0.9300	0.9319	0.9387	0.9378	0.9392	0.9380	0.9390	0.9385	0.9403
UCM	PSNR	29.52	30.73	30.59	30.90	31.10	31.25	31.03	31.20	31.21	31.28
×3	SSIM	0.8135	0.8550	0.8474	0.8565	0.8590	0.8699	0.8575	0.8673	0.8647	0.8677
UCM	PSNR	27.64	28.46	28.31	28.73	28.77	28.85	28.64	28.92	28.77	28.97
×4	SSIM	0.7289	0.7750	0.7619	0.7800	0.7819	0.7841	0.7762	0.7860	0.7803	0.7892

**Table 5:** Per-run PSNR and SSIM results for five independent training runs.

Dataset	Method	Metric	Seed0	Seed1	Seed2	Seed3	Seed4
AID ×4	DCTNet	PSNR	30.994	31.002	30.981	30.988	30.996
		SSIM	0.81772	0.81836	0.81713	0.81725	0.81797
	HAT	PSNR	30.964	30.949	30.962	30.969	30.957
		SSIM	0.81706	0.81684	0.81703	0.81714	0.81695
	MFG-HMoE	PSNR	30.979	30.967	30.972	30.961	30.973
		SSIM	0.81834	0.81829	0.81830	0.81821	0.81837
AID ×3	DCTNet	PSNR	33.234	33.220	33.243	33.219	33.237
		SSIM	0.88064	0.88058	0.88078	0.88050	0.88070
	HAT	PSNR	33.220	33.207	33.232	33.214	33.229
		SSIM	0.88044	0.88069	0.88063	0.88001	0.88042
	MFG-HMoE	PSNR	33.213	33.193	33.211	33.187	33.202
		SSIM	0.87991	0.88059	0.87905	0.88004	0.88027
UCMerced ×4	DCTNet	PSNR	28.974	28.968	28.957	28.989	28.977
		SSIM	0.78920	0.78907	0.78883	0.78931	0.78921
	HAT	PSNR	28.851	28.836	28.845	28.861	28.855
		SSIM	0.78411	0.78374	0.78408	0.78422	0.78404
	MFG-HMoE	PSNR	28.925	28.909	28.918	28.936	28.927
		SSIM	0.78604	0.78588	0.78597	0.78618	0.78608
UCMerced ×3	DCTNet	PSNR	31.283	31.285	31.301	31.277	31.271
		SSIM	0.86770	0.86770	0.86783	0.86763	0.86760
	HAT	PSNR	31.252	31.267	31.231	31.287	31.224
		SSIM	0.86994	0.87012	0.86963	0.87003	0.86885
	MFG-HMoE	PSNR	31.202	31.226	31.198	31.192	31.223
		SSIM	0.86731	0.86772	0.86702	0.86694	0.86737

**Table 6:** Repeated-run results at ×3 and ×4 (mean ± sample SD, n=5).

Dataset	Method	PSNR Mean ± SD	SSIM Mean ± SD
AID ×3	DCTNet	33.231±0.011	0.88064±0.00011
	HAT	33.220±0.010	0.88044±0.00027
	MFG-HMoE	33.201±0.011	0.87997±0.00058
AID ×4	DCTNet	30.992±0.008	0.81769±0.00051
	HAT	30.960±0.008	0.81700±0.00011
	MFG-HMoE	30.970±0.007	0.81830±0.00006
UCMerced ×3	DCTNet	31.283±0.011	0.86769±0.00009
	HAT	31.252±0.026	0.86971±0.00052
	MFG-HMoE	31.208±0.015	0.86727±0.00031
UCMerced ×4	DCTNet	28.973±0.012	0.78912±0.00019
	HAT	28.850±0.010	0.78404±0.00018
	MFG-HMoE	28.923±0.010	0.78603±0.00011

**Table 7:** Welch tests versus the strongest baseline (DCTNet–baseline): *p*-values are Holm-adjusted.

Dataset	Metric	Baseline	Mean Difference	95% CI	Adjusted *p*
AID	PSNR	HAT	+0.0102	[−0.0051,0.0255]	0.3264
×3	SSIM	HAT	+0.000202	[−0.000123,0.000527]	0.3264
AID	PSNR	MFG-HMoE	+0.0218	[0.0109,0.0327]	0.0089
×4	SSIM	MFG-HMoE	−0.000616	[−0.001245,0.000013]	0.2086
UCMerced	PSNR	HAT	+0.0312	[−0.0004,0.0628]	0.2086
×3	SSIM	HAT	−0.002022	[−0.002659,−0.001385]	0.0046
UCMerced	PSNR	MFG-HMoE	+0.0500	[0.0339,0.0661]	<0.001
×4	SSIM	MFG-HMoE	+0.003094	[0.002862,0.003326]	<0.001

**Table 8:** Descriptive class-wise PSNR results on the AID test set for ×4 super-resolution. The best and second-best numerical results are marked in red and blue, respectively. These rankings are descriptive only and do not imply statistical significance.

Class	SRCNN	HSENet	TransENet	SwinIR	ELAN	HAT	DRCT	MFG-HMoE	MambaIRv2-S	Ours
Airport	29.08	30.31	30.46	30.74	30.69	30.78	30.57	30.77	30.73	30.80
Bare land	35.86	37.83	37.99	38.19	37.99	38.20	38.14	38.17	38.20	38.21
Baseball field	31.31	32.73	32.87	33.12	33.05	33.14	32.94	33.10	33.12	33.16
Beach	32.43	33.99	34.03	34.30	34.15	34.32	34.23	34.31	34.32	34.33
Bridge	31.89	32.77	32.92	33.24	33.15	33.25	33.04	33.27	33.23	33.30
Center	27.43	29.04	29.24	29.50	29.55	29.58	29.36	29.63	29.60	29.69
Church	24.38	25.54	25.72	25.93	25.92	25.95	25.76	26.00	25.92	25.98
Commercial	28.01	29.08	29.25	29.45	29.43	29.48	29.31	29.44	29.44	29.50
Dense residential	25.14	26.13	26.31	26.54	26.52	26.57	26.38	26.60	26.53	26.59
Desert	38.32	41.17	41.47	41.59	41.29	41.61	41.55	41.61	41.60	41.62
Farmland	34.13	35.84	35.97	36.22	36.14	36.24	36.06	36.21	36.22	36.26
Forest	29.19	29.84	29.88	29.97	29.95	29.98	29.94	29.95	29.98	29.98
Industrial	26.96	28.22	28.41	28.72	28.69	28.75	28.51	28.78	28.70	28.79
Meadow	33.53	34.29	34.35	34.47	34.40	34.47	34.40	34.47	34.46	34.48
Medium residential	28.12	29.57	29.76	30.02	29.96	30.03	29.85	30.08	29.99	30.03
Mountain	29.93	30.54	30.59	30.70	30.66	30.71	30.66	30.69	30.70	30.72
Park	28.29	29.15	29.26	29.46	29.43	29.47	29.35	29.48	29.45	29.50
Parking	25.02	26.92	27.36	27.97	27.92	28.03	27.43	28.01	27.91	28.06
Playground	29.51	31.33	31.56	31.98	31.90	31.99	31.65	32.00	31.97	32.04
Pond	30.82	31.74	31.80	31.97	31.94	31.98	31.87	32.01	31.95	32.00
Port	26.84	27.89	28.06	28.37	28.34	28.41	28.15	28.37	28.36	28.43
Railway station	28.44	29.71	29.88	30.12	30.08	30.17	29.96	30.20	30.12	30.21
Resort	28.02	29.21	29.37	29.61	29.58	29.64	29.46	29.64	29.61	29.67
River	31.44	32.29	32.34	32.47	32.44	32.48	32.41	32.44	32.47	32.49
School	27.10	28.27	28.43	28.72	28.68	28.75	28.55	28.77	28.71	28.78
Sparse residential	27.17	27.77	27.85	27.97	27.94	27.97	27.89	27.90	27.95	27.97
Square	28.88	30.53	30.69	31.00	30.94	31.03	30.81	31.04	31.00	31.06
Stadium	27.04	28.40	28.57	28.92	28.87	28.95	28.69	29.02	28.90	29.02
Storage tanks	26.24	27.28	27.42	27.63	27.62	27.66	27.48	27.69	27.62	27.69
Viaduct	27.96	29.25	29.42	29.72	29.68	29.73	29.50	29.69	29.69	29.77

**Table 9:** Comparison of model complexity and efficiency for ×4 super-resolution.

Method	Params (M)	FLOPs (G)	FPS	Latency (ms/image)
SwinIR	11.85	202.19	5.54	180.51
HAT	22.51	382.63	5.96	167.79
DRCT	13.72	233.80	3.07	325.73
MFG-HMoE	26.83	441.63	1.27	787.40
MambaIRv2-S	8.92	268.83	7.26	137.74
DCTNet	24.47	399.78	3.38	295.86

## Data Availability

The datasets analyzed in this study are publicly available. The AID and UCMerced datasets can be obtained from the corresponding benchmark release. No new datasets were generated during the current study.
